# Rhinoplasty

**Published:** 1891-03

**Authors:** 


RHINOPLASTY. Being a short description of one hundred cases treated by Tribhovandas Motichand Shah, L. M., Assistant Surgeon and Chief Medical Officer at the Junagadh Hospital. 1889: Printed at the *Junagadh Sarkari Press.*

This interesting book is the record of four years' practice in India with this difficult operation. One hundred cases in four years! This is a number which rarely falls to the lot of a surgeon during a lifetime. It will be asked how does it happen that such a large number of cases should be seen by any one surgeon. The reason is explained in the preface. Outlaws in India do not, as a rule, kill their victims; instead, they cut off their noses. Every act of vengeance for a wrong, real or imaginary, is accomplished by this peculiar species of mutilation. In that country the nose, above all other organs of the body, is considered the organ of respect and reputation. "The usual saying, when a person is told that he has no nose, means that he has forfeited all delicate feelings of honor." A person deprived of his nose is spoken of as a shameless fellow, and looked down upon by society. Such a person is execrated and held as an unfortunate person whose face should seldom be seen. Hence this organ is the target of malice and revenge. Outlaws, called *Makránis,* practice it upon their victims. Husbands inflict it as punishment upon their wives, and upon their wives' paramours. Thus the crime is very common.

There are three methods of making the flap with which the mutilated organ is repaired. One way is from the arms, the second from the cheeks, and the third from the forehead. The author prefers the method of utilizing the integument of the forehead. This method has its drawback in the prominence of the root of the flap, which occurs just at the junction of the forehead with the bridge of the nose. An attempt was made to overcome this deformity by division of the root completely across forty days after the first operation. But after two cases of sloughing, one of which involved the entire flap, this method was abandoned.

The method finally adopted was to dissect down so low as to bring the isthmus of the flap into the oculo-nasal corner, then to unite the *entire* under surface of the flap with the nose. By following this method the prominence is lost. The detail of this operation is given with great minuteness, and is illustrated by diagrams and by photographs of natives taken before and after the operation. W. M. J.

